# A Systematic Review: Role of Pyloric Dilation in the Management of Gastroparesis

**DOI:** 10.1111/nmo.70436

**Published:** 2026-10-21

**Authors:** Milli Gupta, Alberto Barchi, Albert J. Bredenoord, Gwen M. C. Masclee

**Affiliations:** ^1^ Department of Gastroenterology and Hepatology University of Calgary Calgary Alberta Canada; ^2^ Department of Gastroenterology and Gastrointestinal Endoscopy IRCCS San Raffaele Hospital Milan Italy; ^3^ Department of Gastroenterology and Hepatology Academisch Medisch Centrum Amsterdam the Netherlands

**Keywords:** balloon dilation, delayed gastric emptying, gastroparesis, G‐POEM, pneumatic dilation, pyloric dilation

## Abstract

**Background:**

Gastroparesis (GP) is a motility disorder characterized by pronounced delayed gastric emptying and associated with symptoms. Endoscopic pyloric dilation (PyD) is a minimally invasive option targeting pyloric dysfunction, though efficacy remains variably reported.

**Aims:**

This systematic review assessed clinical outcomes and safety of PyD in adults and children with GP not associated with surgical manipulation.

**Methods:**

PubMed, Embase, and Scopus were searched through December 2025 for studies evaluating PyD in GP. Post‐surgical and non‐GP etiologies were excluded. Twelve studies (12 full manuscripts) met inclusion criteria. Data on technique, response rates, and adverse events were extracted.

**Results:**

Quality of studies was fair to poor. Symptom improvement with PyD was described in 37%–85% of treated patients. PyD plus botulin toxin efficacy was reported as 50%–80%. Benefits were reported as early as 2 weeks and up to 2 years after intervention. Repeat dilations were occasionally performed with response ranging from 40% to 95% for adults and children. No serious complications were reported. Adverse events were limited to transient pain reported in one study. Variability in technique and outcome definitions limited pooled analyses.

**Conclusions:**

Within study quality constraints, PyD may be a low‐risk option for symptom relief in select GP patients across all age groups. As there was significant heterogeneity in the data published, well‐designed control studies should be performed to evaluate if this efficacy is convincing. Standardized protocols and integration of novel tools such as EndoFLIP are needed to refine patient selection and assess long‐term efficacy.

## Key Points

- Gastroparesis (GP) is a challenging disorder to treat. Endoscopic pyloric dilation (PyD) is a minimally invasive option targeting pyloric dysfunction, though efficacy remains variably reported.
- A systematic review was completed to assess clinical outcomes and safety of PyD in adults and children with GP not associated with surgical manipulation.
- Symptom improvement with PyD was described in 37%–85% of treated patients. PyD plus botulin toxin efficacy was reported as 50%–80%. Benefits were reported as early as 2 weeks and up to 2 years after intervention. However, study quality is poor and more data are needed to make definitive recommendations.

## Plain Language Summary

Gastroparesis is a difficult condition to treat and medical options are limited. Pyloric dilation involves stretching the pylorus to allow for easy passage of food from the stomach. This may be a low risk option for patients but managing gastroparesis with this has not been critically appraised. This systematic review looked for evidence to use pyloric dilation to treat gastroparesis in adults and children. The systematic review found pyloric dilation reduced symptoms in up to 85% of patients, with benefits lasting over a year. Complications are not reported and most common side effect reported were temporary pain. However, study quality is a limiting factor. Research using new technologies (e.g., EndoFLIP) might help better understand the long term durability and safety of pyloric dilations.

## Graphical abstract

This systematic review highlights the clinical utility of pyloric dilation (PyD) across a range of populations with gastroparesis of varying severities.

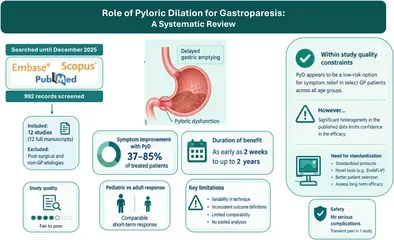

**Abbreviations**
CPGAS: Clinical Patient Grading Assessment Scale
DD: daily diary
DI: distensibility
DM: diameter
GCSI: Gastroparesis Cardinal Symptom Index
GES: gastric emptying study
GIQLI: Gastrointestinal Quality of Life Index
GMA: gastric myoelectrical activity
GP: gastroparesis
G‐POEM: gastric per oral endoscopic myotomy
OBT: octanoic breath test
PAGI‐QOL: patient assessment of upper GI‐quality of life index
PAGI‐SYM: patient assessment of upper GI‐symptoms severity index
PGR: Postprandial Gastric Retention
PS: post surgical
pts: patients
PyD: pyloric dilation
TSS: total symptom score (part of GCSI)

## 1 Introduction

Gastroparesis (GP) is a chronic medical condition characterized by objectively documented delayed gastric emptying in the absence of mechanical obstruction. Symptoms include nausea, post prandial fullness, early satiety, vomiting, bloating, and/or abdominal pain. Diagnosis is confirmed by a combination of symptoms and gastric emptying assessment (nuclear scintigraphy or breath test). GP can clinically be divided into diabetic, post‐surgical, and idiopathic. Typically, dietary and pharmacologic therapies are tried first and include prokinetics (including erythromycin) and antiemetics [1, 2, 3, 4, 5]. However, GP management is a challenge with sparse availability of medications that successfully improve patient symptoms and quality of life. If persistent symptoms debilitate patients, feeding tubes (bypassing the stomach entirely) may be considered to ensure adequate intake.

Pyloric dysfunction is recognized as an important contributor to symptoms of delayed gastric emptying and can be targeted by endoscopic interventions. Targeting the pylorus for improved passage of gastric contents offers the patient a tangible treatment option that mechanistically makes sense. Pylorospasm was identified as a target to treat following a study in the 1980s [6], and invasive interventions with promising results have been published from the use of gastric peroral endoscopic myotomy (G‐POEM) [7]. However, it is not clear whether such treatment can (or should) be applied to all GP. There are concerns with side effects, durability, and the level of invasiveness surgical and endoscopic treatments (like G‐POEM) entail.

Less invasive options like pyloric dilation (PyD) might have a role in managing GP, as it has reported benefits in treating gastric outlet obstruction symptoms in post esophagectomy cases [5, 8]. PyD is recommended by ESGE 2022 in surgical esophagectomy literature [2] but has not been recommended in guidelines for other indications published to date [1, 2, 4]. PyD warrants inquiry given it is relatively easy and safe to implement in clinical practice. The purpose of the systematic review is to summarize the available data on PyD in patients with gastroparesis and appraise the possibility of it as a viable treatment option.

## 2 Methods

The systematic review was undertaken according to the guidelines of the Preferred Reporting Items for Systematic reviews and Meta‐Analyses (PRISMA) checklist [9, 10], following a previously established protocol. The study protocol was registered in the International Prospective Register of Systematic Review (PROSPERO) [10]. No institutional board review approval was needed.

### 2.1 Search Strategy and Study Selection

A systematic literature search was conducted with assistance from the University librarian. All published and unpublished articles on MEDLINE, which include PubMed, Embase, Web of Sciences, and Cochrane databases were investigated using search strings to identify relevant studies. English language articles and abstracts published until December 2025 were eligible for inclusion. All available abstracts were evaluated by two independent reviewers (MG and AB) using Rayyan 2.0 (QDR, Qualitative Data Repository) [11]. Dissents on including studies were resolved after de‐blinding. Hereafter, full texts of the articles were reviewed for inclusion. Publications reported as abstracts were not included as they have not been peer‐reviewed and lack results with clear follow‐up timelines. Differences were resolved by discussion with a third reviewer (GMC).

### 2.2 Eligibility Criteria

Only full text manuscripts were eligible for inclusion. If an abstract and a full text of the same data and institution showed different results, only the full paper was included. Different types of studies were eligible, such as randomized controlled trials, observational studies and case series. Studies had to include patients (both children and adults) with a confirmed diagnosis of GP. The diagnosis was based on two factors—symptoms collected by patient description or validated questionnaires (when available) and gastric emptying study results (GES) (^13^C‐octanoic breath test (OBT) or nuclear scintigraphy). Additional testing such as upper GI endoscopy (with or without food retention after overnight fast) and abdominal imaging (such as CT, ultrasound) were also collected. All studies had to complete GES or mention its performance as a marker of diagnosis for GP. Studies had to report PyD alone or in combination with other treatments (such as botulinum toxin). All types of PyD were included (e.g., pneumatic, esoFLIP, or hydraulic balloon). Exclusion criteria were prior surgery of the esophagus or stomach (e.g., esophagectomy, G‐POEM), bariatric surgery and history of malignancy involving the stomach. GP after fundoplication were included in the study.

### 2.3 Data Collection and Quality Assessment

Demographics of the patients (age, gender, BMI) and study details (e.g., country of origin, study period, study design, patient population) were collected by two authors (M.G. and A.B.). Variables extracted were diagnosis of GP (symptoms, gastric emptying test), change in symptoms recorded by Likert score, or if available by validated questionnaires (such as Gastroparesis Cardinal Symptom Index [GCSI] [12], Patient Assessment of upper Gastrointestinal disorders‐Symptom Severity Index (PAGI‐SYM) [13], Patient Assessment of Upper Gastrointestinal Disorders‐Quality of Life (PAGI‐QOL) [14], Clinical Patient Grading Assessment Scale (CPGAS) [15], GastroIntestinal Quality of Life Index (GIQLI) [16]), etiology of GP, duration of symptoms, prior treatments, definition of clinical success from intervention, type of treatments employed, type of balloon used (including treatment size) and change in gastric emptying rates pre/post intervention [2, 17, 18]. Safety and adverse events related to PyD were also included. Quality of included studies was assessed by using the National Institutes of Health (NIH) quality assessment tool for case series [19]. All reviewers (M.G., A.B., G.M.C.M.) rated each article for quality (good, fair, poor) using the appropriate tools (i.e., separate scales available for case series, RCT, case control) (Tables S1 and S2).

### 2.4 Data Synthesis

Primary outcomes were change in symptoms and objective measurements (gastric emptying tests) after PyD in GP. Secondary outcomes included safety and adverse events, and stratification of the primary outcomes by etiology of gastroparesis, prior treatments (e.g., medications, botulinum toxin), and demographics (e.g., age, gender) of patients studied. For both primary and secondary outcomes, a minimum of two clinical assessments separated by time were required to assess response to intervention. Given the lack of standardized outcome measures and heterogeneity in study populations across the studies, statistical analysis could not be performed.

## 3 Results

### 3.1 Study Selection and Characteristics

After identification of 1651 records and removal of duplicates, a total of 992 records were screened and ultimately 12 studies were included in the systematic review (Figure 1) [21, 22, 23, 24, 25, 26, 27, 28, 29, 30, 31, 32]. Baseline characteristics of the studies are in Table 1. Of the 12 studies, one included adults and children, seven focused on adults, and four on children. Type of studies were diverse (propensity matched observational study, prospective cohort study, prospective case series, prospective case control, and retrospective case series), and majority of studies were single center (*n* = 11).

**FIGURE 1:**
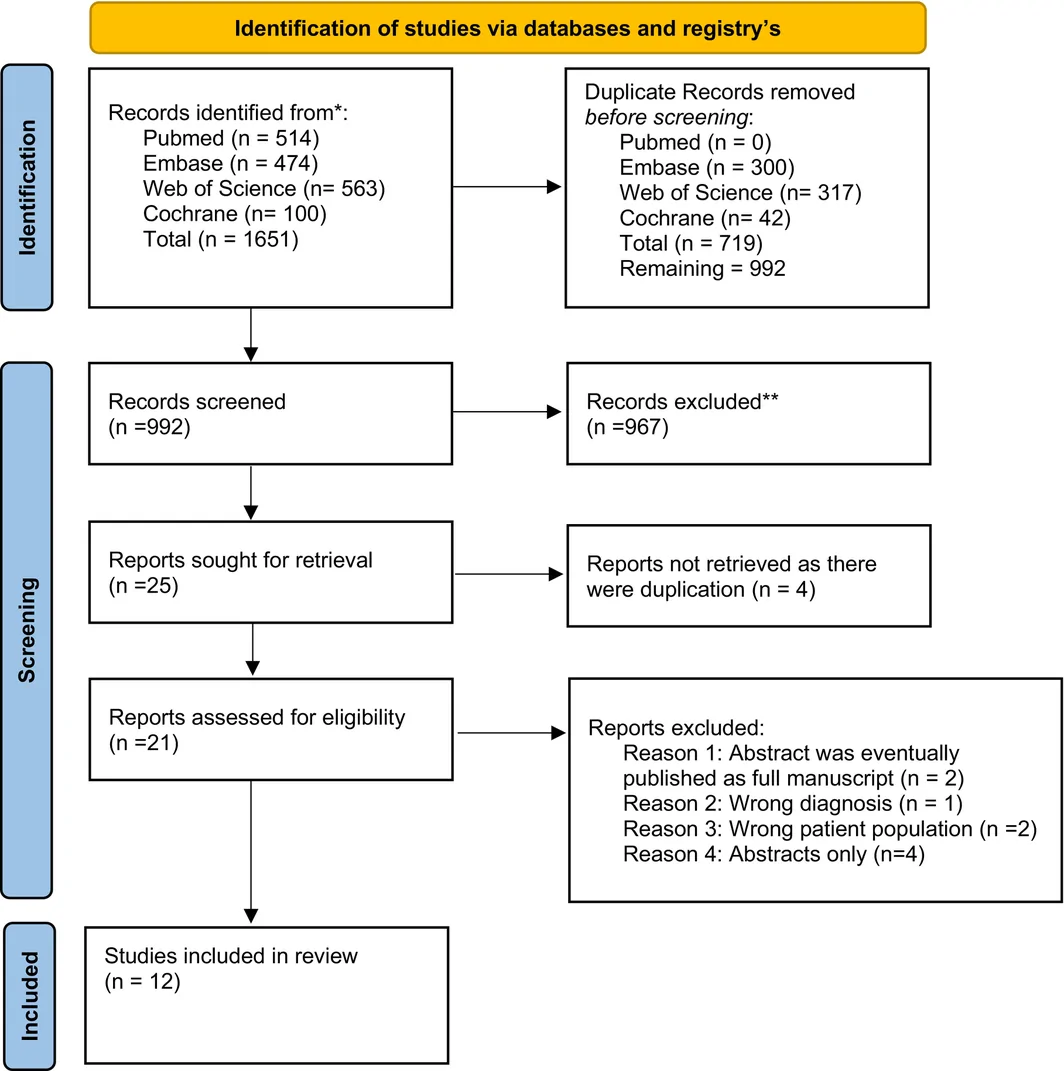
Flow diagram of study selection according to the Preferred Reporting Items for Systematic Reviews and Meta‐analyses (PRISMA) checklist. **Excluded if not meeting inclusion or exclusion criteria, articles not evaluating gastroparesis patients, post‐surgery GP diagnosis, pyloric dilation not included as treatment option, not in English. *From: Page et al. [20].

**TABLE 1:** Study demographics and details of the studies included in systematic review.

Author and year	Study period assessed (years)	Total patients	Patient population	Publication type	Single or multi‐center	Study design	Study quality (NHLBI)	Treatment arms	Dilation type	Male, *n* (%)	Age mean (SD/IQR)
Skarsgard et al. 1998	3	7	Children	Full text	Single	Retrospective case series	Poor	PyD	CRE balloon	—	8.5 (2.5–16)
Israel et al. 2001	2.75	19	Children	Full text	Single	Retrospective case series	Poor	PyD ± surgical pyloroplasty	CRE balloon	11 (58)	3.75y (0.5–10)
Jawaid et al. 2006	11	29	Children	Full text	Single	Retrospective case series	Poor	PyD or surgical pyloroplasty	Pneumatic	18 (62)	1‐16y
Gourcerol et al. 2015	n/a	27	Adults	Full text	Single	Prospective case control	Good	PyD	CRE balloon	12 (44)	42.5y (2.5)
Wellington et al. 2017	11	33	Adults	Full text	Single	Retrospective case series	Fair	PyD or Botox^a^	CRE balloon	3 (9)	42y
Jehangir et al. 2021	1	13	Adults	Full text	Single	Prospective case series	Fair	PyD	CRE balloon	3 (23)	45.2y (5.1)
Mercier et al. 2021	8	24	Children	Full text	Multicenter	Retrospective case series	Fair	PyD or Botox + PyD	CRE balloon	16 (67)	2.7y (4.2)
Murray et al. 2021	2	46	Adults	Full text	Single	Prospective case series	Good	PyD	EsoFLIP dilation	13 (28)	39y (18–88)
Noar et al. 2022	n/a	31	Adults	Full text	Single	Prospective cohort	Fair	PyD	Pneumatic	2 (6.5)	49y (13)
Santucci et al. 2022	7	126	Adults and Children	Full text	Single	Propensity matched observational study	Fair	Botox + PyD	CRE balloon	51 (40)	Control 13.2y (4.7); Cases 14.5y (3.9)
Soliman et al. 2022	3	47	Adults	Full text	Single	Retrospective case series	Fair	PyD	CRE balloon	16 (34)	44
Ericksson et al. 2023	7	204	Adults	Full text	Single	Retrospective case series	Fair	Botox + PyD or PyD alone	Pneumatic	30 (14.7)	51 (36–59)

Abbreviations: NHLBI, National Heart, Lung and Blood Institute; PyD, pyloric dilation (completed with pneumatic, CRE balloon or EsoFLIP).

^a^ Patients in this study had both treatments over the course of the study period but not in same order.

In total, 577 patients with GP were studied (Table 1). Percentage of male patients ranged from 6.5% to 62%. For adults, age range was 18–51 years and for children, 2.7–16 years. Duration of GP symptoms prior to intervention was inconsistently reported: children symptom duration ranged from 6 to 48 months [21, 31] and one mixed population study reported 2 months [24]. One study in adults [30] documented duration of symptoms prior to dilation, but others did not (Table 2a). Follow‐up periods and treatment outcomes are detailed in Table 2a and Table S3.

**TABLE 2:** Definition of Gastroparesis and method of diagnosis.

Author and year	Patient population	Number of patients	Diagnostic criteria for gastroparesis	Baseline mean GES score	Gastroparesis etiology	Symptom duration, mean, months (SD)
Skarsgard et al. 1998	Children	7	T½ > 90 min on GES	6 pts. had *t*½ > 160, and one had *t*½ = 90	Neurologic causes (7)	6–48
Israel et al. 2001	Children	19	T½ > 90 min on GES	32% (8–75)	n/a	n/a
Jawaid et al. 2006	Children	29; 8 PyD	T½ 45–90 min	208 min (67–690); 5 had no results	Cerebral palsy (10), developmental delay (16), post surgical (3)	n/a
Gourcerol et al.2015	Adults	27; 10 PyD	13C‐OBT T½ > 180 min	n/a	Diabetic (4), idiopathic (20), post surgical (3)	n/a
Wellington et al.2017	Adults	33	2 h PGR > 60% or 4 h‐PGR > 10% on GES	70% at 2 h and 42% at 4 h	Diabetic (7), idiopathic (26)	n/a
Jehangir et al. 2021	Adults	13	4 h‐PGR > 10% at GES	35.5% ± 3.5%	Diabetic (2), idiopathic (7), post surgical (2)	3.4 ± 1.5
Mercier et al. 2021	Children	24; PyD alone 12, PyD and botox 6, Botox alone 6	1 h PGR > 90%, 2 h PGR > 60% or 4 h‐PGR > 10% on GES	n/a	Neurologic causes (2), post surgical (5), constipation (4), obesity (1), pyloric stenosis (1)	6
Murray et al. 2021	Adults	46	13C‐octanoic breath test T½ > 180 min	211 min (132–513)	Diabetic (10), idiopathic (33), post surgical (3)	n/a
Noar et al. 2022	Adults	31	T½ > 90 min on GES	33.93% hyper/normal and 37% hypo/normal gastric myoelectrial activity (NS)	Diabetic (4), n/a (27); gastric myoelectrial activity‐hyper/normal (26) and hypo/normal (4)	n/a
Santucci et al. 2022	Adults and children	126	2 h PGR > 60% or 4 h‐PGR > 10% on GES	93.9% controls and 82.65% cases were abnormal	Idiopathic (66), n/a (60)	2.07
Soliman et al. 2022	Adults	47	1 h PGR > 90%, 2 h PGR > 60% or 4 h‐PGR > 10% on GES	n/a	Diabetic (14), idiopathic (14), neurologic causes (4) post surgical (13), chronic intestinal pseudo obstruction (1), infection (1)	n/a
Ericksson et al. 2023	Adults	204; 82 PyD, 46 PD + botox,76 surgery	4 h‐PGR > 10% on GES or T½ > 90 min	n/a	n/a	n/a

Abbreviations: 1 h/2 h/4‐h PGR, percentage gastric retention; CPGAS, Clinical Patient Grading Assessment Scale; DGE, delayed gastric emptying; DI, distensibility; DM, diameter; FTT, failure to thrive; f/up, follow up; GCSI, gastroparesis cardinal symptom index; GES, gastric emptying study; GIQLI, gastrointestinal quality of life index; OBT, octanoic breath test; PAGI‐QOL, patient assessment of upper GI‐quality of life index; PAGI‐SYM, patient assessment of upper GI‐symptoms severity index; PyD, pyloric dilation (completed with pneumatic, CRE balloon, or EsoFLIP); PyS, pyloric stenosis; RCT, randomized controlled trial; TSS, total symptom score (part of GCSI).

### 3.2 Risk of Bias

National Institute of Health quality assessment tool (NHLBI = National Heart, Lung and Blood Institute) was used to evaluate risk of bias for the included studies [19]. Completion of the bias assessment was based on the type of study (e.g., case control, case series, controlled interventional studies, observational cohort) and a ranking form was used to rate each study. Most studies ranked as fair quality [19], with summarized information on study quality in Table S1. Detailed quality assessment results for each study are in Table S2.

### 3.3 Gastroparesis (GP) Definition and Monitoring

Definition of GP was based on gastric emptying study (GES) (^13^C‐octanoic breath test or nuclear scintigraphy) and/or symptoms, but cut offs varied across the studies (Table 2). Two studies [26, 30] defined delayed gastric emptying by GES with Postprandial Gastric Retention of > 10% at 4 h (4 h‐PGR). Four studies [24, 25, 29, 31] used either 1 h PGR cut off > 90%, 2 h PGR > 60%, and/or 4 h PGR > 10%. Four studies [21, 22, 23, 26] defined GP by half‐life of nuclear tag (T½) > 90 min retention in stomach on nuclear scintigraphy; one study [27] considered T½ within 45–90 min to reflect delayed gastric emptying and two [28, 32] diagnosed GP with ^13^C‐OBT T½ > 180 min. Follow‐up was often assessed by symptoms rather than GES, limiting response to intervention analyses (Table S3).

### 3.4 Dilation Protocol and Types

Various pyloric dilators (PyD) were used: hydraulic dilation with controlled radial expansion (CRE) balloon was most common [5] followed by pneumatic [3], and EsoFLIP [1]. None of the studies reported complications with PyD. One study reported transient post procedural pain in 60% of patients after PyD for a median of 1 day [32]. Details on dilator type, size and treatment outcomes are outlined by population in Table 3 (children) and Table 4 (adult). A variety of combinations with PyD were studied: PyD alone [21, 23, 25, 28, 30, 32], PyD plus botulin toxin [24], and PyD and/or botulin toxin [26, 29, 31] PyD and/or surgical pyloroplasty [22, 27]. Some studies used a stepwise approach offering repeat treatments based on patient response (Tables 3 and 4). Protocols for PyD varied, such as rationale for balloon size, type of balloon, and timing of clinical/GES reassessment (2 weeks–21 days). Several studies also allowed adjunct pharmacotherapy, though it was unclear whether these were concurrent with PyD or added after PyD failed.

**TABLE 3:** Pyloric dilation (PyD) protocol in children with GP (5 studies).

Author and year	Treatment arms	Type of dilator	Balloon diameter (mm)	Repeat dilation? (y/n)	Total pts. with PyD, *n*	Pt with repeat PyD, *n* (%)	Definition of treatment success	Partial or complete response to PyD, *n* (%)	Follow up in months, mean (SD)
Skarsgard et al. 1998	PyD	CRE balloon	15 mm for 1–4 years, 18 mm for 5–8 years, 18–20 mm for 9–12 years and 20–25 for > 12 years	y	7	3 (43)	Change in symptoms (no scale), growth chart, GES	5 (71.4%)	4–11
Israel et al. 2001	PyD ± surgical pyloroplasty	CRE balloon	15 mm for < 6 years and 18 mm for > 6 years	y	19	4 (21)	Change in symptoms (no scale), growth chart, FTT status	18 (94.7%)	8.5 for non FTT, 13.4 for FTT pts
Jawaid et al. 2006	PyD or surgical pyloroplasty	Pneumatic	16, 18 or 20–physician dependent	n	8	—	Change in symptoms (no scale) and GES	5 (63%)	n/a
Mercier et al. 2021	PyD or botulin toxin + PyD	CRE balloon	10 mm for < 10 kg; 10–20 mm for 10–30 kg, 20–22 mm for > 30 kg	y	18	—	Change in symptoms, and need for additional treatment	16 (66%)	19.8 (1.7–61.7)
Santucci et al. 2022^a^	Botulin toxin + PyD	CRE balloon	20	n	126	—	Change in symptoms and GES	98 (78%) at 3 months, 99 (79%) at 6 months, 76% at 12 months (propensity matched)	12

Abbreviations: CRE, controlled radial expansion; DI, pyloric distensibility; Dil, dilation; DM, pyloric diameter; f/up, follow up; n, no; pts, patients; PyD, pyloric dilation (completed with pneumatic, CRE balloon, or EsoFLIP); y, yes.

^a^ Mix of adult and children were studied.

**TABLE 4:** Pyloric dilation protocol (PyD) for adults with GP (8 studies).

Author and year	Treatment arms	Type of dilator	Balloon diameter (mm)	Repeat dilation? (y/n)	Total pts. with PyD, *n*	Pt with repeat PyD, *n* (%)	Definition of treatment success	Partial or complete response to PyD, *n* (%)	Follow up in months, mean (SD)
Gourcerol et al. 2015	PyD	CRE balloon	20	n	27	—	Change in GIQLI score and GES. Change in DI on FLIP	10 (37)	1–3
Wellington et al. 2017	PyD and/or botulin toxin^a^	CRE balloon	20	y	33	15 (45)	Change in dominant symptoms and weight	25 (75)	4
Jehangir et al. 2021	PyD	CRE balloon	18, 19, 20	n	13	—	Change in GCSI and CPGAS score. Change in DI and DM on FLIP	5 (38%) at 1 month; 7 (53%) at 3 months; 4 (31%) at 6 months	1, 3 and 6
Murray et al. 2021	PyD	EsoFLIPs	n/a	n	46	—	Change in PAGI‐SYM (including GCSI), PAGI‐QOL, CPGAS score	41 (89)	3.9 (2.2–12.2)
Noar et al. 2022	PyD	Pneumatic	n/a	n	31	—	Change in symptom GCSI‐DD, Leeds dyspepsia and GES by electrical activity	26 (87)	10–12
Santucci et al. 2022^b^	Botulin toxin + PyD	CRE balloon	20	n	126	—	Change in symptoms and GES	98 (78%) at 3 months, 99 (79%) at 6 months, 76% at 12 months (propensity matched)	12
Soliman et al. 2022	PyD	CRE balloon	15, 16.5 and 18	y	47	8 (17)	Change in GCSI score	25 (53%) at 2 months, 19 (40%) at 6 months, 15 (32%) at 2 years	27
Ericksson et al. 2023	Botulin toxin + PyD or PyD alone	Pneumatic	n/a	n	204	—	Change in major symptom severity	47 (57%) PyD alone, 37 (80%) PyD + botulin toxin	0.46

Abbreviations: CPGAS, Clinical Patient Grading Assessment Scale; CRE, controlled radial expansion; DD, daily diary; DI, pyloric distensibility; Dil, dilation; DM, pyloric diameter; f/up, follow up; GCSI, gastroparesis cardinal symptom index; GIQLI, gastrointestinal quality of life index; n, no; PAGI‐QOL, patient assessment of upper GI‐quality of life index; PAGI‐SYM, patient assessment of upper GI‐symptoms severity index; pts, patients; PyD, pyloric dilation (completed with pneumatic, CRE balloon, or EsoFLIP); y, yes.

^a^ Patients in this study had both treatments over the course of the study period but not in same order.

^b^ Mix of adult and children were studied.

In children, one of five studies examined PyD alone [21], two compared PyD with or without surgical pyloroplasty [22, 27], and two examined PyD in combination with botulin toxin [24, 31] (Table 3). Pneumatic dilation was used in one study, and CRE in four studies. Multiple rounds of treatment were used in two studies with benefit [21, 22], but they had different treatment strategies and outcomes (Table 3).

In adults, five of eight studies evaluated PyD alone [23, 25, 28, 30, 32], two studies examined PyD plus botulin toxin [24, 29](Table 4) and one study compared PyD versus PyD combined with botulin toxin [26]. Pneumatic dilation was used in two studies, CRE balloon in five studies and EsoFLIP in one (Table 4). More than one round of treatment was offered in two studies [25, 29] with different interventions. In one study, response to PyD was seen as a predictor of future G‐POEM response [25].

### 3.5 Dilator Balloon Types and Sizes

In children, CRE balloons were most often used with variable size chosen based on patient weight [31], age [21, 22], or size of pylorus relative to the scope [24]. Pneumatic balloons were only used in one study, and the choice of balloon was based on child's size, but details were not provided [27]. In adults, CRE balloons were most often used, with two studies reporting a range of 15–20 mm, but none of them reported a criterion for choosing balloon size [25, 30]. In one study, repeat dilations were allowed [25] and larger diameters were chosen at subsequent dilations. Two studies reported pneumatic dilator use; one reported a goal pressure of 35 psi rather than diameter [23], and the other study did not report either [26]. EsoFLIP dilation [32] required expansion to 25 mm dilation, with a tear response being preferred but not mandatory. Dilation times at the chosen diameter varied from 1 to 2 min across the twelve studies, and endoscopic visualization of a tear or bleeding was not mandatory. Comparative review of efficacy was not possible due to combination treatments, different endpoints and variable timeframes of follow up.

### 3.6 Symptom Based Treatment Success for PyD Versus PyD and/or Botulin Toxin

Four studies evaluated PyD and/or botulin toxin [24, 26, 29, 31] and six studies reported response to PyD alone [21, 23, 25, 28, 30, 32] (Tables 3 and 4). Two studies [24, 29] treated patients with PyD and botulin toxin. One study administered botulin toxin, PyD or both with crossover for repeat treatments [31]. Another study compared PyD vs. combination PyD plus botulin toxin [26].

Change in symptoms was the most common way to report response to PyD across the twelve studies. Symptoms at presentation and enrollment varied across the studies and comprised symptoms typically seen in GP (Table S3). Follow‐up of symptoms was collected in a diverse manner across the cohort, limiting pooled analyses (Table S3). Validated questionnaires used were GCSI [23, 25, 30, 32], GIQLI, CPGAS, Leeds dyspepsia, PAGI‐SYM, and PAGI‐QOL [23, 28, 30, 32] (Table 4). GCSI total scores ranged from 2 to 3.4 at the start of the study, with improvement reported at variable follow‐up times (range 21 days to 12 months). Two studies reported follow‐up from baseline to 6 months [25, 30] but only one study showed ongoing improvement [25] (Table S3). Children had symptoms on a Likert scale with information around nutrition and/or physical metrics collected as endpoints (Table 3). For children, all studies showed improvement but had no standardization post intervention [21, 22, 24, 26, 27, 31] (Table S3).

In adults, partial or complete symptom response from PyD (CRE balloon) alone ranged from 37% to 1 month [28], 75% at 4 months [29], 40% at 6 months [25] to 32% at 27 months [25]. Pneumatic dilator and EsoFLIP reported relatively higher rates of response, noting 87% at 12 months [23] and 85% at 3 months [32] respectively. Combination PyD and botulin toxin with symptomatic relief were similar across time points–80% at 2 weeks [26], 75% at 4 months [29], 76% at 12 months [24] and 66% at 19.8 months [31]. In children, PyD showed symptom response of 71% at 4 months [21] to 94.7% at 13.4 months [22]. PyD and botulin toxin in children showed partial or complete symptom response ranging from 78% to 3 months [24] to 66% response at 19.8 months [31]. Santucci et al. studied both adults and children, but results were provided as a composite summary of the population. This is the only study with a propensity score matched control group that provided both GES and symptom improvement pre and post interventions for the cohort. They found > 80% of patients with delayed GES at baseline had improvement in symptoms by 12 months, compared to 50% of control subjects with no delayed GES (*p* = 0.008). Patients with GP symptoms who had normal or unknown baseline GES documented improvement in symptoms with treatment (70% vs. 65% respectively), but it was not statistically significant compared to controls (*p* = 0.7 and *p* = 0.1 respectively). Cohort with repeat treatment interventions were more likely to achieve higher rates of response ranging from 40% to 94.7% [21, 22, 25, 29, 31].

### 3.7 Outcomes Reported by Duration of Follow Up

Studies reported a range of follow up after intervention, ranging from 2 weeks [26] to 2 years [25]. When categorizing studies based on follow‐up time, three studies reported responses 2 weeks to 3 months after intervention [26, 28, 30]. Seven studies reported treatment response 3 months to 12 months after intervention [21, 22, 23, 24, 29, 30, 32]. Two studies reported outcomes more than 12 months after intervention [25, 31]. Overall, partial or complete response to PyD was noted in 37%–53% treated before 3 months, 53%–95% for 3–12 months, and 32%–76% at > 12 months. The higher responders were more likely to be in the cohort that had repeated intervention (botulin toxin or surgical pyloroplasty) with PyD (Table S3, Tables 3 and 4).

Some studies provided outcome assessment at multiple time points of follow up (e.g., 1, 3, and 6 months or 3, 6, or 12 months). Two studies in adult patients looked at PyD alone in such a manner. One study noted better symptom response at the second follow up (6 months) compared to earlier timepoints [30] but this effect was not observed in the second study [25]. Repeated checks over a longer period were not reported for children, except in the Santucci et al. [24, 26] study that had a mix of both populations. Trends for response after PyD and botulin toxin noted static responses reported at 3, 6, and 12 months in adults and children (Tables 3 and 4).

### 3.8 Gastric Emptying Assessment After Pyloric Dilation

In adults, GES testing after PyD was reported in three studies. One [23] showed worsening of gastric emptying time in patients with lower than normal gastric myoelectrical activity (GMA), whereas gastric emptying improved in patients with normal GMA (baseline T½ < 90 min increased from 33.9% to 72% after intervention). Another study [28] showed improved gastric emptying with 13C‐OBT from 287 ± 37 min at baseline to 224 ± 29 min. Mixed population study [24] noted improvement in GES from baseline in those with GP compared to controls (81% vs. 50%; *p* = 0.008) (Table 4). In children, four studies reported gastric emptying time in follow‐up [21, 22, 24, 27] and showed improvement from baseline testing after PyD (and/or botulin toxin) (Table S3). Due to variability in the metrics reported, time of reassessment and type of treatments completed, pooled analyses were not possible.

## 4 Discussion

This systematic review highlights the clinical utility of pyloric dilation (PyD) across a range of populations with GP of varying severities. There was a wide range in follow up, study quality, and study types in the literature. Despite this, most studies in this review demonstrated symptoms and/or GES improvement with PyD in combination with adjunct therapies like botulin toxin. PyD is a relatively minimally invasive option, with no significant adverse events or complications reported in over 577 patients from 1998 to 2022 in this literature review.

One of the challenges in making definitive conclusions is the heterogeneity in quality and type of the studies in the literature. Santucci et al. [24] is the only study with a placebo‐controlled arm in this review, underscoring the need for more rigorous trials to decide on PyD's role (with or without botox) in GP. In the methodology of defining gastroparesis, there was significant variability of tests and thresholds used to diagnose GP, as well as a lack of validated and clinically accessible method to assess pyloric function (only one study used FLIP). Regarding the technique of PyD, studies reported inconsistent dilation techniques, diameters, and variable timeframes of follow‐up to determine treatment efficacy. There was also a range of symptom scoring tools with variable outcome assessment timelines and post‐intervention gastric emptying testing reported. These are just some of the difficulties encountered in summarizing data in this review. Despite these concerns, the usage of PyD reflects real‐world variation on GP diagnosis and management. There was a constellation of etiologies of GP studied, and PyD did not have a trend of improvement in one type over another: suggesting applicability in various settings.

All studies required gastric emptying assessment for the diagnosis of gastroparesis. However, it is possible studies may have included functional dyspepsia‐predominant patients in their cohort. Gastric emptying test results and symptoms do not always correlate with each other, and it is possible patients have GP and functional dyspepsia overlap with variable gastric emptying rates over the lifespan of the disease [33]. This overlap could have contributed to reduced efficacy of PyD. In keeping with guidelines, symptoms and GES results were used to identify GP patients. Perhaps FLIP can help bridge this gap, but literature is still uncertain on the benchmarks of abnormal pylorus diameter (DM) and distensibility (DI) to employ, as well as exact placement in the diagnostic process [34]. If studies provided pre/post‐treatment GES values (like Santucci et al.) and combined it with pyloric DM and DI measurements (like Jehangir et al.), this perhaps would facilitate identifying the populations best served by PyD. More study is required and highlights the unmet needs in gastroparesis.

Another potential role for PyD is diagnostic; to pre‐screen patients suitable for invasive procedures like surgical pyloroplasty or gastric per oral myotomy (G‐POEM) [35]. In one study reviewed, PyD responders were more likely to respond to G‐POEM compared to nonresponders (71% vs. 37.5%; *p* = NS) [26]. While this finding was not statistically significant, PyD could possibly screen for subsequent G‐POEM responders. The study also followed patients up to 2 years after treatment, one of the longest follow up data with PyD, establishing a signal for durability by PyD alone. Perhaps PyD can be used post G‐POEM to augment response in those that show recurrence, though the data are limited and not definitive [34]. In another study [26], response to PyD alone did not indicate future response to pyloroplasty or G‐POEM. Therefore, care needs to be taken when considering the impact and response to the manipulation of pylorus and gastric emptying.

PyD may have synergistic benefits in combination with botulin toxin. PyD in conjunction with botulin toxin showed generally favorable results. Wellington et al. [29] noted treatments (botulin toxin and PyD) applied twice may be associated with higher improvement in symptoms and associated weight gain, but this was not statistically significant. While it is not possible to compare the data head‐to‐head, patients with all types of GP did relatively well with PyD plus botulin toxin compared to PyD. Patient's response differed based on timeline of follow up, so care must be taken when considering the results. Adults and children with PyD and botulin toxin were more often reported as having symptomatic response. With PyD alone, response rates were higher in children (71%–95%) than adults (27%–53%) with CRE balloon. Pneumatic dilators and EsoFLIP were not used in more than one study, but given their mechanism of dilation involves higher pressures, it lends to reason they result in more robust response as reported here. However, the true physiologic effect is difficult to interpret due to inconsistent reporting of gastric emptying response with PyD. Also, it is difficult to draw conclusions from symptom assessments given variable tools used and inconsistent timelines of follow up.

Combination PyD and botulin toxin have also been evaluated for its diagnostic role in pre‐screening for G‐POEM. Eriksson et al. [27] assessed the impact of PyD + botulin toxin vs. PyD alone on future G‐POEM or pyloroplasty response. They noted patients with botulin toxin intervention alone were 3 times more likely to respond to surgical intervention compared to PyD alone, regardless of patient symptom response and GP etiology. The use of botulin toxin is not recommended by clinical guidelines, as greater benefits were not reported when compared against sham treatments. Another reason for caution is the concern that repeated use might induce inflammatory and fibrotic changes in the pylorus [3, 36, 37]. Similar concerns are also raised with PyD, where recurrent tears in the region might result in fibrosis. This reinforces the importance of a more physiological based approach to treating GP. It is theoretically possible that combination treatment with PyD alters the pyloric diameter and improves gastric accommodation and/or antral‐duodenal motor coordination. Pyloric distensibility has been shown to change with botulin toxin in GP patients when evaluated on FLIP, with associated benefit in GIQLI, symptoms, and GES results [38]. Distensibility change has been reported with PyD alone [28]. Using PyD balloon combined with botulin toxin before considering more invasive interventions with G‐POEM may allow physicians to provide an individualized approach to GP treatment. Combination therapies (e.g., PyD + botulin toxin) show promise but the lack of long‐term data, standardized outcome measures and robust control groups limit conclusions and warrant prudence around its application in the clinical setting.

There were limited publications looking at types of PyD balloon in GP. Pneumatic dilation and CRE balloon can likely be used for GP management in children and adults. In children, the size of CRE balloon was based on age or weight, but the cutoffs employed were variable. In adults, the CRE balloon diameter most often used was 20 mm, but there was no clear duration of treatment or documentation of endoscopically visible response to verify efficacy. Assessment of durability of dilation was limited by inconsistent timelines employed in studies. Reports [8] identified a lower number of repeat dilations with pneumatic versus CRE balloon in pyloric stenosis, with no increase in adverse events. While this might be of value to study to better define PyD's safety profile, our review did not identify high rates of complications. Either type of PyD may be a reasonable first step for children and patients for whom surgical or endoscopic myotomy may be contraindicated.

The recent AGA guidelines listed more recommendations against use of medications than for use in GP [4]. This highlights an unmet need in care of GP patients. Our review highlights the potential benefit PyD could deliver. A nuanced study of PyD, however, is needed to make an informed decision on how and if to position PyD in GP care. For example, should medications still be used as a first‐line approach despite limited benefit and extensive side effect profiles? While no adverse events were reported in the studies evaluated in this review of PyD, heterogeneity of data quality in conjunction with limited durability of symptom response and lack of gastric emptying results after PyD suggests we must remain critical of making sweeping changes in treatment algorithms for GP. Most guidelines do not discuss PyD, except for ESGE, which identified PyD for post‐esophagectomy cases only [3]. Most studies provide short‐ to mid‐term follow‐up (1–12 months) with PyD, with only one retrospective study reporting 2 years follow up [25], highlighting that durability is another aspect that warrants attention.

There were limitations to this review. Patterns observed across studies—despite methodological variability—offer important preliminary signals that can guide hypothesis generation and future trial design. Similar challenges were identified in post fundoplication dysphagia, where retrospective uncontrolled series identified reasonably good efficacy for pneumatic dilation, but a prospective, randomized, sham controlled clinical trial demonstrates no efficacy [39]. Given the paucity of high‐quality evidence, exclusion of studies with poor or fair methodology would have precluded meaningful synthesis. There was a trend for positive response with PyD in treating a variety of etiologies of GP. PyD might be adaptable to different clinical settings and GP symptom profiles, but caution must be exercised when considering its application in clinical care.

In conclusion, pyloric dilation may be beneficial to treat medically refractory gastroparesis in both children and adults. Symptom improvements presented across multiple studies should be interpreted with caution given the lack of controlled data and risk of bias. Pyloric dilation may have utility as a therapeutic option and as a diagnostic tool to identify those candidates best suited and responsive to advanced and invasive pylorus treatments like G‐POEM. It may also have a role in combination with botulin toxin. However, robust randomized placebo‐controlled trials are essential to determining the true efficacy of pyloric dilation in gastroparesis.

## Author Contributions

Conceptualization, Milli Gupta: Gwen M.C. Masclee and Albert J. Bredenoord: methodology. Milli Gupta: Gwen M.C. Masclee: Alberto Barchi and Albert J. Bredenoord: review and statistical analysis. Milli Gupta and Alberto Barchi: writing – original draft preparation. Milli Gupta: Alberto Barchi: Gwen M.C. Masclee and Albert J. Bredenoord: writing – review and editing. Milli Gupta: Alberto Barchi: Gwen M.C. Masclee and Albert J. Bredenoord: supervision. Gwen M.C. Masclee. All authors have read and agreed to the published version of the manuscript.

## Conflicts of Interest

M.G.—received speaker and/or consulting fees from Sanofi/Regeneron, AstraZeneca, Takeda, Eupraxia, and AVIR pharma Inc. Research funding from Celgene, BMS, Dr. Falk, Eupraxia, and Medtronic. A.B.‐none to declare. A.J.B. received research funding from Sanofi/Regeneron, Uniquity, Aqilion, Laborie, and Dr. Falk Pharma and received speaker and/or consulting fees from AlfaSigma, Apogee, Uniquity, Laborie, BMS, Dr. Falk Pharma, Eupraxia, Aqilion, Alimentiv, Sanofi/Regeneron, Reckitt, Domain, and AstraZeneca. G.M.C.M.‐none to declare.

## Supporting information


**Table S1:** Summary of 16 studied included in analysis were evaluated using the National Institutes of Health (NIH) quality assessment tool, tailored to evaluate the type of study completed (e.g., case series, observational cohort, no control group, controlled intervention studies).
**Table S2:** Individual quality assessment forms for each study reviewed for the systematic review quality assessment tool for case series studies.

**Table S3:** Treatment responses captured in the 12 studies (symptoms and GES).

## Data Availability

The data that support the findings of this study are available from the corresponding author upon reasonable request.
