# Supplementary material for: A Systematic Review: Role of Pyloric Dilation in the Management of Gastroparesis
Source: Neurogastroenterol Motil. 2026 Oct 21;38(10):e70436. doi: 10.1111/nmo.70436 (PMC13687569; doi:10.1111/nmo.70436)
Supplement: Supplementary file 1 [file NMO-38-e70436-s001.docx]

Supplementary Table 1 - Summary of 16 studied included in analysis were evaluated using the National Institutes of Health (NIH) quality assessment tool, tailored to evaluate the type of study completed (for example, case series, observational cohort, no control group, controlled intervention studies).

| **Author and Year** | **Study design** | **Treatment  arms** | **Study quality (NHLBI)** |
| --- | --- | --- | --- |
| Skarsgard et al. 1998 | Retrospective case series | PyD | Poor |
| Israel et al. 2001 | Retrospective case series | PyD +/- surgical pyloroplasty | Poor |
| Jawaid et al. 2006 | Retrospective case series | PyD or surgical pyloroplasty | Poor |
| Gourcerol et al. 2015 | Prospective case control series | PyD | Good |
| Wellington et al. 2017 | Retrospective case series | PyD or Botox^ | Fair |
| Jehangir et al. 2021 | Prospective case series | PyD | Fair |
| Mercier et al. 2021 | Retrospective case series | PyD or Botox or Botox + PyD | Fair |
| Murray et al. 2021 | Prospective case series | PyD | Good |
| Noar et al. 2022 | Prospective case series | PyD | Fair |
| Santucci et al. 2022 | Retrospective case series | Botox + PyD | Fair |
| Soliman et al. 2022 | Retrospective case series | PyD | Fair |
| Eriksson et al. 2023 | Retrospective case series | Botox + PyD or PyD alone | Fair |

Supplementary Table 2: Individual quality assessment forms for each study reviewed for the systematic review

Quality assessment tool for case series studies

**Skarsgard et al., 1998**

| **Criteria** | **Yes** | **No** | **Other (CD, NR, NA)*** |
| --- | --- | --- | --- |
| 1. Was the study question or objective clearly stated? | Y |  |  |
| 2. Was the study population clearly and fully described, including a case definition? | Y |  |  |
| 3. Were the cases consecutive? |  |  | CD |
| 4. Were the subjects comparable? |  | N |  |
| 5. Was the intervention clearly described? | Y |  |  |
| 6. Were the outcome measures clearly defined, valid, reliable, and implemented consistently across all study participants? |  | N |  |
| 7. Was the length of follow-up adequate? |  |  | NR |
| 8. Were the statistical methods well-described? |  |  | NA |
| 9. Were the results well-described? |  | N |  |

**Quality Rating (Good, Fair, or Poor)**

Rater #1 initials: GM

Rater #2 initials: **AB**

Additional Comments (If POOR, please state why):

Poor (no validated questionnaires used, no standard follow-up considered in all subjects. Unclear if cases were consecutive or not, no outcome measures, old gp definition

*CD, cannot determine; NA, not applicable; NR, not reported

Quality assessment tool for case series studies

**Israel et al., 2001**

| **Criteria** | **Yes** | **No** | **Other (CD, NR, NA)*** |
| --- | --- | --- | --- |
| 1. Was the study question or objective clearly stated? | Y |  |  |
| 2. Was the study population clearly and fully described, including a case definition? | Y |  |  |
| 3. Were the cases consecutive? |  |  | NR |
| 4. Were the subjects comparable? |  |  | CD |
| 5. Was the intervention clearly described? | Y |  |  |
| 6. Were the outcome measures clearly defined, valid, reliable, and implemented consistently across all study participants? |  | N |  |
| 7. Was the length of follow-up adequate? | Y |  |  |
| 8. Were the statistical methods well-described? |  | N |  |
| 9. Were the results well-described? | x |  |  |

**Quality Rating (Good, Fair, or Poor)**

Rater #1 initials: GM

Rater #2 initials: **AB**

Additional Comments (If POOR, please state why): **POOR**

*CD, cannot determine; NA, not applicable; NR, not reported

Quality assessment tool for case series studies

**Jawaid et al., 2006**

| **Criteria** | **Yes** | **No** | **Other (CD, NR, NA)*** |
| --- | --- | --- | --- |
| 1. Was the study question or objective clearly stated? |  | N |  |
| 2. Was the study population clearly and fully described, including a case definition? |  | N |  |
| 3. Were the cases consecutive? |  | N |  |
| 4. Were the subjects comparable? |  |  | CD, probably yes |
| 5. Was the intervention clearly described? | Y |  |  |
| 6. Were the outcome measures clearly defined, valid, reliable, and implemented consistently across all study participants? |  | N |  |
| 7. Was the length of follow-up adequate? |  |  | NR |
| 8. Were the statistical methods well-described? |  |  | NR |
| 9. Were the results well-described? |  | N |  |

**Quality Rating (Good, Fair, or Poor)**

Rater #1 initials: GM

Rater #2 initials: **AB**

Additional Comments (If POOR, please state why): **POOR**

*CD, cannot determine; NA, not applicable; NR, not reported

Quality Assessment Tool for **Before-After (Pre-Post) Studies With No Control Group** (second review of same paper)

**Jawaid et al., 2006**

| **Criteria** | **Yes** | **No** | **Other (CD, NR, NA)*** |
| --- | --- | --- | --- |
| 1. Was the study question or objective clearly stated? |  | N |  |
| 2. Were eligibility/selection criteria for the study population prespecified and clearly described? |  | N |  |
| 3. Were the participants in the study representative of those who would be eligible for the test/service/intervention in the general or clinical population of interest? |  | N |  |
| 4. Were all eligible participants that met the prespecified entry criteria enrolled? |  |  | CD |
| 5. Was the sample size sufficiently large to provide confidence in the findings? |  |  | CD |
| 6. Was the test/service/intervention clearly described and delivered consistently across the study population? |  | N |  |
| 7. Were the outcome measures prespecified, clearly defined, valid, reliable, and assessed consistently across all study participants? |  | N |  |
| 8. Were the people assessing the outcomes blinded to the participants' exposures/interventions? |  | N |  |
| 9. Was the loss to follow-up after baseline 20% or less? Were those lost to follow-up accounted for in the analysis? |  |  | CD |
| 10. Did the statistical methods examine changes in outcome measures from before to after the intervention? Were statistical tests done that provided p values for the pre-to-post changes? |  | N |  |
| 11. Were outcome measures of interest taken multiple times before the intervention and multiple times after the intervention (i.e., did they use an interrupted time-series design)? |  | N |  |
| 12. If the intervention was conducted at a group level (e.g., a whole hospital, a community, etc.) did the statistical analysis take into account the use of individual-level data to determine effects at the group level? |  |  | NA |

**Quality Rating (Good, Fair, or Poor) (see guidance)**

Rater #1 Initials: GM

Rater #2 Initials: **AB**

Additional Comments (If POOR, please state why): **POOR**

Quality Assessment Tool for **Observational Cohort and Cross-Sectional Studies-Goucerol et al., 2015**

| **Criteria** | **Yes** | **No** | **Other (CD, NR, NA)*** |
| --- | --- | --- | --- |
| 1. Was the research question or objective in this paper clearly stated? | Y |  |  |
| 2. Was the study population clearly specified and defined? | Y |  |  |
| 3. Was the participation rate of eligible persons at least 50%? | Y |  |  |
| 4. Were all the subjects selected or recruited from the same or similar populations (including the same time period)? Were inclusion and exclusion criteria for being in the study prespecified and applied uniformly to all participants? | Y |  |  |
| 5. Was a sample size justification, power description, or variance and effect estimates provided? |  |  | NA |
| 6. For the analyses in this paper, were the exposure(s) of interest measured prior to the outcome(s) being measured? | Y |  |  |
| 7. Was the timeframe sufficient so that one could reasonably expect to see an association between exposure and outcome if it existed? | Y |  |  |
| 8. For exposures that can vary in amount or level, did the study examine different levels of the exposure as related to the outcome (e.g., categories of exposure, or exposure measured as continuous variable)? |  |  | NA |
| 9. Were the exposure measures (independent variables) clearly defined, valid, reliable, and implemented consistently across all study participants? | Y |  |  |
| 10. Was the exposure(s) assessed more than once over time? |  |  | NA |
| 11. Were the outcome measures (dependent variables) clearly defined, valid, reliable, and implemented consistently across all study participants? | Y |  |  |
| 12. Were the outcome assessors blinded to the exposure status of participants? |  | N |  |
| 13. Was loss to follow-up after baseline 20% or less? |  |  | NR |
| 14. Were key potential confounding variables measured and adjusted statistically for their impact on the relationship between exposure(s) and outcome(s)? | Y |  |  |

**Quality Rating (Good, Fair, or Poor)**

**GOOD**

Rater #1 initials: GM

Rater #2 initials: **AB**

Additional Comments (If POOR, please state why): **GOOD**

Quality Assessment Tool for **Before-After (Pre-Post) Studies With No Control Group**

**Wellington et al., 2017**

| **Criteria** | **Yes** | **No** | **Other (CD, NR, NA)*** |
| --- | --- | --- | --- |
| 1. Was the study question or objective clearly stated? | Y |  |  |
| 2. Were eligibility/selection criteria for the study population prespecified and clearly described? | Y |  |  |
| 3. Were the participants in the study representative of those who would be eligible for the test/service/intervention in the general or clinical population of interest? | Y |  |  |
| 4. Were all eligible participants that met the prespecified entry criteria enrolled? | Y |  |  |
| 5. Was the sample size sufficiently large to provide confidence in the findings? |  |  | CD |
| 6. Was the test/service/intervention clearly described and delivered consistently across the study population? | Y |  |  |
| 7. Were the outcome measures prespecified, clearly defined, valid, reliable, and assessed consistently across all study participants? | Y |  |  |
| 8. Were the people assessing the outcomes blinded to the participants' exposures/interventions? |  | N |  |
| 9. Was the loss to follow-up after baseline 20% or less? Were those lost to follow-up accounted for in the analysis? |  |  | CD |
| 10. Did the statistical methods examine changes in outcome measures from before to after the intervention? Were statistical tests done that provided p values for the pre-to-post changes? |  | N |  |
| 11. Were outcome measures of interest taken multiple times before the intervention and multiple times after the intervention (i.e., did they use an interrupted time-series design)? |  | N |  |
| 12. If the intervention was conducted at a group level (e.g., a whole hospital, a community, etc.) did the statistical analysis take into account the use of individual-level data to determine effects at the group level? |  |  | NA |

**Quality Rating (Good, Fair, or Poor) (see guidance)**

Rater #1 Initials: MG

Rater #2 Initials: **AB**

Additional Comments (If POOR, please state why): **FAIR**

Quality Assessment Tool for **Before-After (Pre-Post) Studies With No Control Group**

**Jehangir et al., 2021**

| **Criteria** | **Yes** | **No** | **Other (CD, NR, NA)*** |
| --- | --- | --- | --- |
| 1. Was the study question or objective clearly stated? | Y |  |  |
| 2. Were eligibility/selection criteria for the study population prespecified and clearly described? | Y |  |  |
| 3. Were the participants in the study representative of those who would be eligible for the test/service/intervention in the general or clinical population of interest? | Y |  |  |
| 4. Were all eligible participants that met the prespecified entry criteria enrolled? | Y |  |  |
| 5. Was the sample size sufficiently large to provide confidence in the findings? |  |  | CD |
| 6. Was the test/service/intervention clearly described and delivered consistently across the study population? | Y |  |  |
| 7. Were the outcome measures prespecified, clearly defined, valid, reliable, and assessed consistently across all study participants? | Y |  |  |
| 8. Were the people assessing the outcomes blinded to the participants' exposures/interventions? |  | N |  |
| 9. Was the loss to follow-up after baseline 20% or less? Were those lost to follow-up accounted for in the analysis? |  | N |  |
| 10. Did the statistical methods examine changes in outcome measures from before to after the intervention? Were statistical tests done that provided p values for the pre-to-post changes? | Y |  |  |
| 11. Were outcome measures of interest taken multiple times before the intervention and multiple times after the intervention (i.e., did they use an interrupted time-series design)? | Y |  |  |
| 12. If the intervention was conducted at a group level (e.g., a whole hospital, a community, etc.) did the statistical analysis take into account the use of individual-level data to determine effects at the group level? |  |  | NA |

**Quality Rating (Good, Fair, or Poor) (see guidance)**

Rater #1 Initials: MG

Rater #2 Initials: **AB**

Additional Comments (If POOR, please state why): **FAIR**

Quality Assessment Tool for **Before-After (Pre-Post) Studies With No Control Group**

**Mercier et al., 2021**

| **Criteria** | **Yes** | **No** | **Other (CD, NR, NA)*** |
| --- | --- | --- | --- |
| 1. Was the study question or objective clearly stated? | Y |  |  |
| 2. Were eligibility/selection criteria for the study population prespecified and clearly described? |  | N |  |
| 3. Were the participants in the study representative of those who would be eligible for the test/service/intervention in the general or clinical population of interest? | Y |  |  |
| 4. Were all eligible participants that met the prespecified entry criteria enrolled? |  |  | CD |
| 5. Was the sample size sufficiently large to provide confidence in the findings? |  |  | CD |
| 6. Was the test/service/intervention clearly described and delivered consistently across the study population? | Y |  |  |
| 7. Were the outcome measures prespecified, clearly defined, valid, reliable, and assessed consistently across all study participants? |  | N |  |
| 8. Were the people assessing the outcomes blinded to the participants' exposures/interventions? |  | N |  |
| 9. Was the loss to follow-up after baseline 20% or less? Were those lost to follow-up accounted for in the analysis? | Y |  |  |
| 10. Did the statistical methods examine changes in outcome measures from before to after the intervention? Were statistical tests done that provided p values for the pre-to-post changes? |  | N |  |
| 11. Were outcome measures of interest taken multiple times before the intervention and multiple times after the intervention (i.e., did they use an interrupted time-series design)? |  | N |  |
| 12. If the intervention was conducted at a group level (e.g., a whole hospital, a community, etc.) did the statistical analysis take into account the use of individual-level data to determine effects at the group level? |  |  | NA |

**Quality Rating (Good, Fair, or Poor) (see guidance)**

Rater #1 Initials: MG

Rater #2 Initials: **AB**

Additional Comments (If POOR, please state why): **FAIR**

Quality Assessment Tool for **Before-After (Pre-Post) Studies With No Control Group**

**Murray et al., 2021**

| **Criteria** | **Yes** | **No** | **Other (CD, NR, NA)*** |
| --- | --- | --- | --- |
| 1. Was the study question or objective clearly stated? | Y |  |  |
| 2. Were eligibility/selection criteria for the study population prespecified and clearly described? | Y |  |  |
| 3. Were the participants in the study representative of those who would be eligible for the test/service/intervention in the general or clinical population of interest? | Y |  |  |
| 4. Were all eligible participants that met the prespecified entry criteria enrolled? | Y |  |  |
| 5. Was the sample size sufficiently large to provide confidence in the findings? | Y |  |  |
| 6. Was the test/service/intervention clearly described and delivered consistently across the study population? | Y |  |  |
| 7. Were the outcome measures prespecified, clearly defined, valid, reliable, and assessed consistently across all study participants? | Y |  |  |
| 8. Were the people assessing the outcomes blinded to the participants' exposures/interventions? |  | N |  |
| 9. Was the loss to follow-up after baseline 20% or less? Were those lost to follow-up accounted for in the analysis? | Y |  |  |
| 10. Did the statistical methods examine changes in outcome measures from before to after the intervention? Were statistical tests done that provided p values for the pre-to-post changes? | Y |  |  |
| 11. Were outcome measures of interest taken multiple times before the intervention and multiple times after the intervention (i.e., did they use an interrupted time-series design)? |  | N |  |
| 12. If the intervention was conducted at a group level (e.g., a whole hospital, a community, etc.) did the statistical analysis take into account the use of individual-level data to determine effects at the group level? |  |  | NA |

**Quality Rating (Good, Fair, or Poor) (see guidance)**

Rater #1 Initials: MG

Rater #2 Initials: **AB**

Additional Comments (If POOR, please state why): **GOOD**

Quality Assessment Tool for **Observational Cohort and Cross-Sectional Studies**

**Noar et al., 2022**

| **Criteria** | **Yes** | **No** | **Other (CD, NR, NA)*** |
| --- | --- | --- | --- |
| 1. Was the research question or objective in this paper clearly stated? | Y |  |  |
| 2. Was the study population clearly specified and defined? | Y |  |  |
| 3. Was the participation rate of eligible persons at least 50%? |  |  | CD |
| 4. Were all the subjects selected or recruited from the same or similar populations (including the same time period)? Were inclusion and exclusion criteria for being in the study prespecified and applied uniformly to all participants? | Y |  |  |
| 5. Was a sample size justification, power description, or variance and effect estimates provided? |  | N |  |
| 6. For the analyses in this paper, were the exposure(s) of interest measured prior to the outcome(s) being measured? | Y |  |  |
| 7. Was the timeframe sufficient so that one could reasonably expect to see an association between exposure and outcome if it existed? | Y |  |  |
| 8. For exposures that can vary in amount or level, did the study examine different levels of the exposure as related to the outcome (e.g., categories of exposure, or exposure measured as continuous variable)? |  |  | NA |
| 9. Were the exposure measures (independent variables) clearly defined, valid, reliable, and implemented consistently across all study participants? | Y |  |  |
| 10. Was the exposure(s) assessed more than once over time? | Y |  |  |
| 11. Were the outcome measures (dependent variables) clearly defined, valid, reliable, and implemented consistently across all study participants? | Y |  |  |
| 12. Were the outcome assessors blinded to the exposure status of participants? |  | N |  |
| 13. Was loss to follow-up after baseline 20% or less? | Y |  |  |
| 14. Were key potential confounding variables measured and adjusted statistically for their impact on the relationship between exposure(s) and outcome(s)? | Y |  |  |

**Quality Rating (Good, Fair, or Poor)**

Rater #1 initials: GM

Rater #2 initials: **AB**

Additional Comments (If POOR, please state why): **GOOD**

Quality Assessment **of Case-Control Studies**

**Santucci et al., 2022**

| **Criteria** | **Yes** | **No** | **Other (CD, NR, NA)*** |
| --- | --- | --- | --- |
| 1. Was the research question or objective in this paper clearly stated and appropriate? | x |  |  |
| 2. Was the study population clearly specified and defined? | x |  |  |
| 3. Did the authors include a sample size justification? |  | x |  |
| 4. Were controls selected or recruited from the same or similar population that gave rise to the cases (including the same timeframe)? |  |  | CD |
| 5. Were the definitions, inclusion and exclusion criteria, algorithms or processes used to identify or select cases and controls valid, reliable, and implemented consistently across all study participants? |  |  | CD |
| 6. Were the cases clearly defined and differentiated from controls? |  | x | GP pt can be identified |
| 7. If less than 100 percent of eligible cases and/or controls were selected for the study, were the cases and/or controls randomly selected from those eligible? |  |  | CD |
| 8. Was there use of concurrent controls? |  |  | CD |
| 9. Were the investigators able to confirm that the exposure/risk occurred prior to the development of the condition or event that defined a participant as a case? |  | x |  |
| 10. Were the measures of exposure/risk clearly defined, valid, reliable, and implemented consistently (including the same time period) across all study participants? |  | x |  |
| 11. Were the assessors of exposure/risk blinded to the case or control status of participants? |  | x |  |
| 12. Were key potential confounding variables measured and adjusted statistically in the analyses? If matching was used, did the investigators account for matching during study analysis? | x |  |  |
|  |  |  |  |
| **Quality Rating (Good, Fair, or Poor) (see guidance)** |  |  |  |
| Rater #1 Initials: GM |  |  |  |
| Rater #2 Initials: MG |  |  |  |
| Additional Comments (If POOR, please state why): FAIR. Table 3 shows results for those with or without DGE. Can only extrapolate results from those patients with confirmed DGE. Cases are those with balloon dilation and botulin toxin vs controls with conservative management |  |  |  |

Quality Assessment Tool for **Before-After (Pre-Post) Studies With No Control Group**

**Soliman et al., 2022**

| **Criteria** | **Yes** | **No** | **Other (CD, NR, NA)*** |
| --- | --- | --- | --- |
| 1. Was the study question or objective clearly stated? | Y |  |  |
| 2. Were eligibility/selection criteria for the study population prespecified and clearly described? | Y |  |  |
| 3. Were the participants in the study representative of those who would be eligible for the test/service/intervention in the general or clinical population of interest? | Y |  |  |
| 4. Were all eligible participants that met the prespecified entry criteria enrolled? |  |  | NR |
| 5. Was the sample size sufficiently large to provide confidence in the findings? |  |  | CD |
| 6. Was the test/service/intervention clearly described and delivered consistently across the study population? | Y |  |  |
| 7. Were the outcome measures prespecified, clearly defined, valid, reliable, and assessed consistently across all study participants? | Y |  |  |
| 8. Were the people assessing the outcomes blinded to the participants' exposures/interventions? |  | N |  |
| 9. Was the loss to follow-up after baseline 20% or less? Were those lost to follow-up accounted for in the analysis? | Y |  |  |
| 10. Did the statistical methods examine changes in outcome measures from before to after the intervention? Were statistical tests done that provided p values for the pre-to-post changes? | Y |  |  |
| 11. Were outcome measures of interest taken multiple times before the intervention and multiple times after the intervention (i.e., did they use an interrupted time-series design)? |  | N |  |
| 12. If the intervention was conducted at a group level (e.g., a whole hospital, a community, etc.) did the statistical analysis take into account the use of individual-level data to determine effects at the group level? |  |  | NA |

**Quality Rating (Good, Fair, or Poor) (see guidance)**

Rater #1 Initials: GM

Rater #2 Initials: **AB**

Additional Comments (If POOR, please state why): **Fair**

Quality Assessment Tool for **Before-After (Pre-Post) Studies With No Control Group**

**Eriksson et al., 2023**

| **Criteria** | **Yes** | **No** | **Other (CD, NR, NA)*** |
| --- | --- | --- | --- |
| 1. Was the study question or objective clearly stated? | Y |  |  |
| 2. Were eligibility/selection criteria for the study population prespecified and clearly described? | Y |  |  |
| 3. Were the participants in the study representative of those who would be eligible for the test/service/intervention in the general or clinical population of interest? | Y |  |  |
| 4. Were all eligible participants that met the prespecified entry criteria enrolled? |  |  | CD |
| 5. Was the sample size sufficiently large to provide confidence in the findings? |  |  | CD |
| 6. Was the test/service/intervention clearly described and delivered consistently across the study population? | Y |  |  |
| 7. Were the outcome measures prespecified, clearly defined, valid, reliable, and assessed consistently across all study participants? | Y |  |  |
| 8. Were the people assessing the outcomes blinded to the participants' exposures/interventions? |  | N |  |
| 9. Was the loss to follow-up after baseline 20% or less? Were those lost to follow-up accounted for in the analysis? |  |  | NA |
| 10. Did the statistical methods examine changes in outcome measures from before to after the intervention? Were statistical tests done that provided p values for the pre-to-post changes? | Y |  |  |
| 11. Were outcome measures of interest taken multiple times before the intervention and multiple times after the intervention (i.e., did they use an interrupted time-series design)? |  | N |  |
| 12. If the intervention was conducted at a group level (e.g., a whole hospital, a community, etc.) did the statistical analysis take into account the use of individual-level data to determine effects at the group level? |  |  | NA |

**Quality Rating (Good, Fair, or Poor) (see guidance)**

Rater #1 Initials: GM

Rater #2 Initials: **AB**

Additional Comments (If POOR, please state why): **FAIR.** Not good based on no consecutive patients, no initial prior documentation of selection criteria, power, exposure.
