# Supplementary material for: A Systematic Review: Role of Pyloric Dilation in the Management of Gastroparesis
Source: Neurogastroenterol Motil. 2026 Oct 21;38(10):e70436. doi: 10.1111/nmo.70436 (PMC13687569; doi:10.1111/nmo.70436)
Supplement: Supplementary file 2 [file NMO-38-e70436-s002.docx]

| **Author and Year** | **Patient (Pt) population** | **Treatment**  **arms** | **Pt in study** | **Self reported symptoms pretreatment** | **Dominant symptom reported** | **Symptom questionnaires, if used** | **Follow up in months, mean (SD)** | **Definition of treatment success** | **Symptom resolution or improvement after PyD, n (%)** | **Details of results and response** | **Indication for Post treatment** **GES test** | **Post treatment** **GES results (n, % population)** |
| --- | --- | --- | --- | --- | --- | --- | --- | --- | --- | --- | --- | --- |
| Skarsgard et al. 1998 | Children | PyD | 7 | Vomiting (no scores), FTT, growth chart | vomiting | n/a | 4-11 | Change in symptoms (no scale), growth chart, GES | 5 (71.4%) | n/a | Improvement from baseline | GES normal (t½ <160min) (4, 57%) |
| Israel et al. 2001 | Children | PyD +/- surgical pyloroplasty | 19 | Delayed vomiting, early satiety, FTT, growth chart | n/a | n/a | 8.5 for non FTT, 13.4 for FTT pts | Change in symptoms (no scale), growth chart, FTT status | 18 (94.7%) | n/a | Incomplete response | Mean score 27% (4, 21%) |
| Jawaid et al. 2006 | Children | PyD or surgical pyloroplasty | 29; 8 PyD | Upper GI symptoms | n/a | n/a | n/a | Change in symptoms (no scale) and GES | 5 (62.5%) | n/a | Improvement from baseline | 2 normal and 2 improved (4, 50%, no scores) |
| Gourcerol et al. 2014 | Adults | PyD | 27; 10 PyD | Upper GI symptoms | n/a | GIQLI score, FLIP measures | 1-3 | Change in GIQLI score and GES. Change in DI on FLIP | Not provided | PyD in 10 GP pts with low fasting DI (<10 mm²/mmHg)  showed increased DI from 7.4 to 20.1 mm²/mmHg. (P < 0.01). Improved GIQLI scores from 72.5+/- 5.5 to 89.3 +/- 6.1 (p=0.04).  Pyloric DI associated with reduced QOL, reduced GES emptying and more symptom burden | All pts offered, 8 tested | T½ improved in 7/8 pts from 287 +/-37 min to 224 +/- 29 min (p= 0.15) in pts with low fasting DI (<10).  (8, 80%). |
| Wellington et al. 2017 | Adults | PyD or Botox† | 33 | Nausea (79%), vomiting (n=15%), epigastric fullness and early satiety (n = 6%). | Nausea was the major symptom in 76% of patients | Symptoms adapted from GCSI (no score) | 4 | Change in dominant symptoms and weight | 75% (25/33 pts), 72% (18/25 pts), and 88% (13/17 pts) improved after first, second, and third treatment | Average weight gain of 1.54lbs (p<0.04) with range of -12 to +29 lbs | n/a | n/a |
| Jehangir et al. 2021 | Adults | PyD | 13 | n/a | n/a | GCSI score, FLIP measures | 1, 3 and 6 | Change in GCSI and CPGAS score. Change in DI and DM on FLIP | All (100%) GCSI TSS improvement at 1 month (p=0.04). Trends for improvement in bloating subscale (p=0.06) and upper abdominal pain subscale (p=0.05). At 3-months, n/v subscale improved (p<0.05) but not TSS p=0.06. At 6 months, bloating and upper abdo discomfort (individual symptoms) improved p<0.05 but not TSS p=0.07. | Improvement in CPGAS was significant at 1- and 3-month f/up (p<0.01). On exclusion of those with gastric stimulator, improvement at 6-months was significant (p<0.05).  FLIP DI lower in symptom responders compared to non responders at 30ml (4.7 vs. 13.2, p=0.04) at 40ml (7.2 vs 13.9, p=0.02) and 50ml (4.9 vs. 10.2, p=0.035). Pyloric DM did not show a difference. | n/a | n/a |
| Mercier et al. 2021 | Children | PyD or Botox or Botox + PyD | 24; PyD alone 12, PyD and botox 6, Botox alone | Feeding type (oral, EN, TPN), GI symptoms, weight, height, and BMI | n/a | n/a | 19.8 (1.7-61.7) | Change in symptoms, and need for additional treatment | 16 (66%). Efficacy and recurrence did not differ between arms.  Multiple treatments completed in 63% of pts. | 87% tolerate oral feeds. Drug treatment prescribed in 52%. | Symptomatic | Ongoing abnormality (no details) (5, 27.8%) |
| Murray et al. 2021 | Adults | PyD | 46 | n/a | n/a | PAGI-SYM, PAGI-QOL, CPGAS | 3.9 (2.2-12.2) | Change in PAGI-SYM (including GCSI), PAGI-QOL, CPGAS score | 39 (85%) completed assessment at 21 days, and 28 (61%) completed assessment at 3.9 months. | PAGI-SYM improved from 2.45 to 2.05 (21d) to 1.65 (3.9 mon) (p<0.001). The GCSI improved from 2.78 to 2.44 (21 days) to 1.95 (3.9 mon) (p<0.01).  PAGI-QOL improved from 1.92 to 1.43 (21d) to 1.27 (3.9 mon) (p=0.020).  CPGAS improved in 64% at 21d and 57% at 3.9 months | All patients | Mean 179 minutes (SD 88-441). (41, 89%) |
| Noar et al. 2022 | Adults | PyD | 31 | Early satiety (100%), bloating (79%), fullness (66%), upper abdominal discomfort (66%) and  discomfort worsened by eating (93%), nausea (55%) | n/a | Leeds dyspepsia, GCSI -DD (daily diary) score | 10-12 | Change in symptom scores and GES by electrical activity (GMA): Group 1 (hyper/normalGMA) and group 2 (hypo/normal GMA) | 26 (87%) | Leeds and GCSI-DD had positive change in all symptoms in group 1 (p<0.001), and no change in group 2 (p>0.05) | All patients | Improved from 33.93 to 72% in group 1 (p<0.001). Worse from 37% to 29.5% in group 2 (p<0.001). (30, 96.7%) |
| Santucci et al. 2022 | Adults and Children | Botox + PyD | 126 | Abdominal pain (78%), nausea (52%), vomiting (47%), early satiety (8%), weight loss (6.5%). Psychiatric comorbidities present in 41% | n/a | n/a | 12 | Change in symptoms and GES | 98 (78%) at 3 months, 99 (79%) at 6 months, 76% at 12 months (propensity matched to controls) | Nausea and vomiting responded most favorably (p=NS). Age <14, males, delayed GES at baseline and no psychiatric comorbidities responded favorably (p=NS) | All patients | Those with delayed GES at baseline improved (81%) compared to controls (50%) (p=0.008). (31, 24.6%) |
| Soliman et al. 2022 | Adults | PyD | 47 | n/a | n/a | GCSI score | 27 | Change in GCSI score | 10 (21%) had sustained response to 1 PyD treatment at 2 years. Total 15 (31.9%) responded in 2 years. | GCSI improved at 2 months (p<0.0001) and sustained to 6 months. No predictor of clinical response | n/a | n/a |
| Ericksson et al. 2023 | Adults | Botox + PyD or PyD | 204; 82 PyD, 46 PD + botox,76 surgery | Major GI symptom | Vomiting, bloating, abdominal pain, other | n/a | 0.46 | Change in major symptom severity | 47/82 (57%) with PyD alone, and 37/46 (80%) PD + botox had symptom response (no details). | PD alone was inferior to PD + botox by a factor of 3. GP etiology had no impact on outcome | n/a | n/a |

Supplementary Table 3: Treatment responses captured in the twelve studies (symptoms and GES).

Legend: CPGAS = Clinical Patient Grading Assessment Scale, DI = distensibility, DM= diameter, f/up = follow up, GES= Gastric Emptying study, GCSI = Gastroparesis cardinal symptom index, GIQLI =Gastrointestinal Quality of Life Index, PAGI-SYM= Patient Assessment of Upper GI -Symptoms Severity Index, PAGI-QOL = Patient Assessment of Upper GI - Quality of Life Index, PS = post surgical, pts = patients, PyD=pyloric dilation (completed with pneumatic, CRE balloon, or EsoFLIP), TSS = total symptom score (part of GCSI); 1h/2h/4-hr PGR = Percentage Gastric Retention, †= patients in this study had both treatments over the course of the study period but not in same order.
